# Impact of Cognitive Impairment on Functional Outcome in Stroke

**DOI:** 10.4061/2010/652612

**Published:** 2010-03-11

**Authors:** Nurdan Paker, Derya Buğdaycı, Demet Tekdöş, Betül Kaya, Çağlayan Dere

**Affiliations:** Istanbul Physical Medicine and Rehabilitation Training Hospital, 2nd PMR Clinic, Istanbul 34180, Turkey

## Abstract

The aim of this study was to investigate the effect of the cognitive impairment on functional status in patients with subacute stroke. Fifty-two patients with subacute stroke were included in the study. Mini mental state examination (MMSE) test was used for the evaluation of cognitive status. Patients were separated into two groups according to their cognitive functions. Functional follow-up parameters were activities of daily living (ADL), global recovery and ambulation status. All patients were evaluated on admission to rehabilitation unit, at discharge and 6 months after discharge. Forty-four patients were completed the study. Mean age was 66 and 57 years; disease duration on admission was 4,8 and 3,5 months in the cognitively impaired and normal groups, respectively. Significant improvement was found in terms of functional follow-up parameters in both groups at discharge (*P* < .05). Functional follow-up parameters did not show statistically significant difference between the groups. But community ambulation rate was higher in cognitively normal group at the sixth month visit. As a result of this study, inpatient rehabilitation was effective both cognitively normal and impaired subacute stroke patients.

## 1. Introduction

Stroke may cause physical and cognitive impairments. Age, functional status and disease duration on admission, co-morbidities, and cognitive functions are known to be the predictors of functional outcome in stroke [1–6]. 

Acute poststroke cognitive impairment is commonly seen [7, 8]. Cognitive impairment occurs in 35.2%–43.9% of the patients three months after stroke and may continue for a long time in approximately 1/3 of the patients [9–11]. 

Cognitive impairment may lead decrease in functional capacity, therefore it affects rehabilitation outcomes in stroke [12]. Some investigators reported that cognitive impairment might have negative effect on functional outcome and activities of daily living (ADL) [10, 13–15]. Stephens et al. reported that mild global cognitive impairment and mild attention loss had negative effect on ADL in the older stroke survivors [13]. Zinn et al. concluded that improvement rate of more complex life activities as measured by Lawton instrumental ADL score was lower among the stroke patients with cognitive impairment but cognitive impairment did not have negative effect on recovery of ADL [16]. Cognitive impairment did not completely block the efficacy of rehabilitation. A recent study showed that acute stroke patients with cognitive impairment had significant functional gain after rehabilitation intervention [17]. 

As another parameter in this study, community ambulation is a valuable follow-up measure because of decreased physical capacity and impairment in cognitive functions may interfere with walking ability after stroke [18]. Community ambulation might be influenced by several factors including walking speed, motor function, balance, endurance, and using a walking aid [19]. Kollen et al. suggested that standing balance was more important in walking recovery than the strengthening of paralytic lower extremities [20]. 

The purpose of this study was to investigate the effect of poststroke cognitive impairment on ADL, ambulation, and global recovery after inpatient rehabilitation and 6-month follow-up period.

## 2. Methods

Fifty-two patients with subacute stroke admitted to the rehabilitation unit between 01.10.2004–28.02.2006 were included in this study. Diagnosis was made according to the World Health Organization (WHO) stroke definition by a neurologist.

Exclusion criteria were aphasia, insufficient communication skills, visual loss, unconsciousness, and having vascular demans or Alzheimer's disease diagnoses before stroke. Cranial MRI or CT was used for diagnosing the etiology of stroke.

According to the MMSE total scores, patients were divided into two groups. First group was consisted of cognitively impaired patients (MMSE < 21). Cognitively normal patients (MMSE ≥ 22) were included in the second group.

## 3. Measurements

### 3.1. MMSE

MMSE is a simple, practical, and reliable test that is used for the assessment of the cognitive functions [21]. Cognitive functions are evaluated at the following six areas: orientation, memory, attention, calculation, language, and construction functions. Total score changes between 0–30. Although MMSE is frequently used for evaluating cognitive functions in clinical practice, it has some limitations. It is concluded that MMSE could be inadequate in evaluating mild forms of cognitive dysfunction and cognitive impairments due to right hemisphere dysfunction. Age and education status may also influence MMSE results [22]. MMSE scores have been shown statistically significant correlation with education level [23, 24]. MMSE cutoff score changes according to the education status of the individuals. Mean MMSE score was found 22 for the patients with 0–4 years education in a population-based study [23]. The reliability and validity of Turkish version of MMSE has been studied [25].

### 3.2. Modified Barthel Index (BI)

BI helps to evaluate 10 different areas of ADL: feeding, transfers, grooming, toilet use, bathing, mobility, stair climbing up and down, dressing, bowel and bladder control [26]. Total score changes between 0–100. Higher score shows better performance in ADL.

### 3.3. Modified Rankin Scale

Modified Rankin Scale is an easy and reliable test which is used for the evaluation of global outcome. The test has six stages. Zero indicates asymptomatic persons. Stages 1 and 2 show minimal symptoms and mild disability. Stages 3, 4 and, 5 describe moderate and severe disability. Modified Rankin Scale is one of the most frequently used tests for the assessment of activity limitation [27].

### 3.4. Ambulation

Functional ambulation was assessed at four levels. (1) Nonambulatory (2) Nonfunctional ambulation (3) Household ambulation, and (4) Community ambulation.

All patients had inpatient rehabilitation in stroke unit. Rehabilitation program was consisted of Bobath and Proprioceptive Neuromuscular Facilitation (PNF) exercises, balance and walking training performed by two physiotherapists. All patients participated in the same rehabilitation program 5 days a week in the stroke unit. Home exercise program was planned individually at discharge. The neurologist who assessed the cognitive functions of the patients did not have an effect on determining discharge time of the patients. Patients were evaluated at admission to the rehabilitation unit, at discharge and 6 months after discharge. In this study all patients were discharged home. The hospital ethics committee approved this study.

Statistical analysis was performed by using SPSS for Windows version 10.0. Chi-square test, Paired *t*, test and Wilcoxon test were used for descriptive analysis and for comparisons in the groups. Student *t* test and Mann-Whitney *U* test were used for the comparison of the groups.

Repeated measures analysis of variance test was used for evaluation. *P*-value <.05 was considered as statistically significant. 

## 4. Results

Clinical characteristics of the patient groups are summarized in Table 1. There were 28 (63,6%) cognitively impaired and 16 (34,4%) cognitively normal patients. Mean age was statistically significantly higher in cognitive impairment group (*P* < .05). Education level was significantly lower in cognitively impaired patients (*P* < .05). Stroke-related data of the patient groups are shown in Table 2. Disease duration on admission and LOS did not show a statistically significant difference between groups. Forty-four patients completed the study. At the sixth-month follow-up period 4 of the patients died and 4 lost to follow-up. Two of the patients died and 3 lost to follow-up in cognitive impairment group. Two patients died and 1 lost to follow-up in cognitive intact group. 

Significant recovery occurred in terms of Barthel scores, global recovery and ambulation status at discharge and continued during the 6-month follow-up in both groups (Table 3) (*P* < .05). There was no significant difference between the groups in terms of BI scores, ambulation status and modified Rankin scores on admission to the stroke unit and at discharge (*P* > .05). Ambulation level was improved significantly during the sixth-month follow-up (*P* < .05), however, Barthel scores and modified Rankin scores did not show statistically significant improvement by the end of the study according to discharge values (*P* > .05).

**Figure 1:**
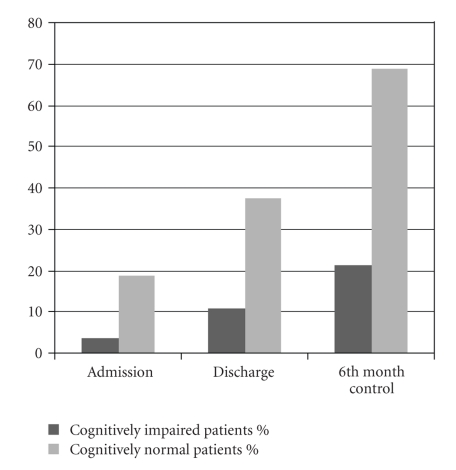
Community ambulation rates of the patients.

Figure 1 shows higher community ambulation rate among the cognitively normal patients. Community ambulation rates were 3,6% (*n* = 1) and 18,8% (*n* = 3) on admission in the cognitively impaired and cognitively normal groups, respectively. Community ambulation rates were increased to 21,4% (*n* = 6) and 68,8% (*n* = 11) at the end of the study in the cognitively impaired and cognitively normal groups, respectively.

## 5. Discussion

Functional status as evaluated by modified Barthel index, modified Rankin scale, and ambulation level showed significant improvement after inpatient rehabilitation in stroke patients with and without cognitive impairment in this study. Rabadi et al. concluded that significant functional gain, as evaluated by Functional Independence Measurement (FIM) occurred in cognitively intact and cognitively impaired acute stroke patients [17]. According to the results of another study, rehabilitation might lead to significant functional recovery in the patients with cognitive impairment [28].

Significant improvement in functional ambulation after inpatient rehabilitation and increase in community ambulation rate during the sixth-month follow-up were important findings in this study. It is concluded that walking ability might have been improved in the first year after stroke in a previous study [29]. The improvement in walking ability is more significant in the early poststroke period and improvement rate might decrease by time [20].

Improvement of ambulation in cognitively normal and impaired groups did not show statistically significant difference, but community ambulation rate was higher in cognitively normal group. Community ambulation frequency was lower among the cognitively impaired patients also at the baseline and at the sixth-month visit. More than 2/3 of the cognitively normal stroke patients were doing community ambulation; however, this rate was only 1/5 for the cognitively impaired persons. Standing balance might influence ambulation; however, other factors are necessary for building community ambulation. It is concluded that combining the walking ability with other factors such as cognitive and behavioral functions is necessary to achieve the community ambulation [18]. In this study the factors that may affect community ambulation like standing balance, motor functions, endurance, walking speed, and using walking aids were not evaluated; however, low education level and older age in cognitive impairment group might have negative effect on community ambulation.

A previous study showed that although rehabilitation intervention was successful in patients with cognitive impairment, functional capacity was low at discharge [12].

As a result of this study ADL and global recovery showed significant improvement in both stroke groups after inpatient rehabilitation. ADL and global recovery did not show difference after discharge in the study groups. Zinn et al. concluded that cognitive impairment had no negative effect on functional improvement as evaluated by FIM in a group of postacute stroke patients [16].

Functional outcome scores of the cognitive impairment group at admission to inpatient rehabilitation unit was lower than those cognitively normal patients' in this study; however, there was no statistically significant difference between the groups in terms of functional parameters. Diamond et al. concluded that the reason of the poor functional outcome among the cognitively impaired geriatric patients was the low functional status at admission to rehabilitation unit [12].

Length of stay (LOS) in cognitively impaired and normal groups did not show difference in this study. This result is consistent with a previous study conducted on geriatric rehabilitation patients [12]. Some other previous studies showed that stroke patients with severe cognitive impairment had a longer LOS [17, 28]. Yu and Richmond stated that cognitive impairment does not lead to decrease in the efficacy of outpatient rehabilitation, and has no effect on the treatment duration and functional recovery in the elderly [30].

Previous study results are contradictory on the subjects with cognitive impairment having negative effect on rehabilitation intervention and functional outcome in stroke due to the different features of patients like age, disease duration on admission to rehabilitation and severity of cognitive impairment. Because of the patients in this study were not very old and had mostly mild to moderate cognitive impairment, rehabilitation program might become successful. Relatively small patient group, failure of MMSE test in evaluating all of cognitive deficits, and exclusion of the patients with severe cognitive deficits like aphasics were the limitations of this study. In this study 6 month follow-up after discharge was valuable for predicting functional outcome.

In conclusion, inpatient rehabilitation program was successful in patients with and without cognitive impairment in this study. Cognitive dysfunction interfered with community ambulation in patients with stroke, but did not have a significant effect on ADL and global recovery. Being aware of cognitive impairment in stroke patients might be useful for predicting the functional prognosis and future planning. 

## Figures and Tables

**Table 1:** Clinical characteristics of the patients.

	Cognitive impairment group	Cognitively normal group	*P*-value
Number of the patients *n* (%)	28 (63.6)	16 (34.4)	
Age (years)	66.2 ± 9.7	57.7 ± 12.6	.01*
Gender			
Female *n* (%)	21 (75)	10 (62.5)	
Male *n* (%)	7 (25)	6 (37.5)	.08^†^
Profession			
Housewife *n* (%)	20 (71.4)	5 (31.3)	
Retired *n* (%)	5 (17.9)	9 (56.2)	.00^†^
Employee *n* (%)	3 (10.7)	2 (12.5)	
Education (years)	1.61 ± 2,92	6.69 ± 3.89	.00*

**Table 2:** Stroke related characteristics of the patient groups.

	Cognitive impairment group	Cognitively normal group	*P*-value
MMSE total scores	16.82 ± 4.41	26.81 ± 2.5	.00*
Etiology			
Ischemia *n* (%)	19 (67.9)	12 (75.0)	.00^†^
Haemorrhage *n* (%)	9 (32.1)	4 (25.0)	
Hemiplegic side			
Right *n* (%)	13 (46.4)	7 (44.8)	>.05^†^
Left *n* (%)	15 (53.6)	9 (56.2)	
Stroke duration (mos)	4.82 ± 2.89	3.56 ± 3.01	>.05*
LOS (days)	35.96 ± 8.14	36,81 ± 5,50	>.05*

**Table 3:** Barthel Index scores, ambulation levels and Modified Rankin Stages of the study groups.

	Cognitively impaired group			*P* value	Cognitively normal group			*P* value
	Admission	Discharge	6. Month Control		Admission	Discharge	6. Month Control	
Barthel Index score	52.32 ± 20.83	61.25 ± 18.44	65.89 ± 18.41	.000* <.05**	60.94 ± 22.15	72.19 ± 22.11	71.56 ± 17.5	.000* <.05^∗ ∗^
Ambulation Level	1.79 ± 0.91	2.71 ± 0.81	2.82 ± 0.90	<.004^†^ <.05**	2.13 ± 1.25	3.13 ± 0.88	3.44 ± 0.96	<.000^†^ <.05**
Modified Rankin Stage	3.46 ± 0.92	3.07 ± 0.90	2.93± 0.97	<.000^†^ <.05**	3.13 ± 1.20	2.81± 0.98	2.81 ± 1.1	<.00^†^ <.05**

*variance analysis for repetitive measurements

^†^friedman test

**paired *t* test.
